# Integrated bioinformatics data analysis reveals a risk signature and PKD1 induced progression in endometrial cancer patients with postmenopausal status

**DOI:** 10.18632/aging.204168

**Published:** 2022-07-09

**Authors:** Yun Cheng, Suyun Zhang, Yan Qiang, Lingyan Dong, Yujuan Li

**Affiliations:** 1 Department of Gynecology, Nanjing First Hospital, Nanjing Medical University, Nanjing 210000, Jiangsu Province, China

## Abstract

Background: Endometrial cancer (EC) is one of the most common type of female genital malignancies. The purpose of the present study was to reveal the underlying oncogene and mechanism that played a pivotal role in postmenopausal EC patients.

Methods: Weighted gene co-expression network analysis (WGCNA) was conducted using the microarray dataset and clinical data of EC patients from The Cancer Genome Atlas (TCGA) and Gene Expression Omnibus (GEO) databases to identify significant gene modules and hub genes associated with postmenopausal status in EC patients. LASSO regression was conducted to build and validate the risk model. Finally, expression of hub gene was validated in pre- and post-menopausal EC patients in our center.

Results: 1240 common genes were used to construct the WGCNA model. According to the WGCNA results, we identified a brown module with 471 genes which was significantly associated with postmenopausal status in EC patients. Furthermore, we constructed an 11-gene risk signature to predict the overall survival of EC patients. The Kaplan–Meier curve and area under the ROC curve (AUC) of this model showed high accuracy in prediction. We also validate the risk model in patients in our center and it also has a high accuracy. Among the 11 genes, PKD1 was recognized as a potential biomarker in the progression of EC patients with postmenopausal status.

Conclusion: Taken together, we uncovered a common PKD1-mediated mechanism underlying postmenopausal EC patients’ progression by integrated analyses. This finding may improve targeted therapy for EC patients.

## INTRODUCTION

Endometrial carcinoma (EC) is a common female malignant tumor, which has increasingly become a burden in the world and China [1]. In the past decade, the number of Chinese women diagnosed with EC has increased significantly, which may be attributed to higher rates of obesity, diabetes, hypertension, aging, early menarche, and late menopause [2]. EC comes from proliferative endometrium (type I, endometrioid) or atrophic endometrium (type II, non-endometrioid), which are related to estrogen [3]. However, most of the women diagnosed with EC are postmenopausal women aged 60 to 70, and 56% of women with type I were diagnosed after menopause, which indicates that estrogen is not decisive risk factor for patients with type I EC [4, 5]. There is an urgent need to study its mechanism underlying postmenopausal women with type I EC and to discover new prognostic molecular biomarkers.

Advancements in high-throughput microarray technology have made it possible to identify genes associated with EC progress using gene expression profiling [6], though studies using these techniques have assessed differentially expressed genes (DEGs) and have not considered the relationship between them. In these cases, genes with similar patterns of expression could be associated with each other. There are limitations in selecting DEGs only between normal and cancer samples, and we should pay more attention to the association between gene expression and clinical characteristics. Weighted gene co-expression network analysis (WGCNA) is commonly used to characterize the relationships between genes and can identify associations that are highly correlated [7]. WGCNA would classify genes with similar functions in the same module by gene expression profiling, and summarize the identified modules by the module eigengene, relating eigengene network to one another and to clinical features. It has been commonly used to identify hub genes in the following cancers: clear cell renal cell carcinoma (ccRCC) [8], pancreatic ductal adenocarcinoma [9], and breast cancer [10].

Due to the limitation of experiment, the development of huge public transcriptome database provides an excellent platform for cancer research, screening biomarkers associated with prognosis and clinicopathological characteristics [11]. Machine learning methods have been developed using RNA-sequencing patterns, which can be used to develop models for accurate classification and prediction in medical settings. Different subtypes of EC are quite different in terms of molecular characteristics and treatments [12].

In the current study, we hoped to reveal the potential mechanism of tumorigenesis in postmenopausal women with type I EC. We constructed a correlation network of DEGs from publicly accessible resources by WGCNA, and identified a gene module that had a close association with postmenopausal status. Furthermore, risk model was constructed and validated in TCGA dataset. Finally, we analyzed the PKD1 expression levels in tissues from patients with premenopausal or postmenopausal status and explored the mechanical value of PKD1 in EC patients.

## MATERIALS AND METHODS

### Data collection

The Cancer Genome Atlas (TCGA) UCSC XENA (https://xena.ucsc.edu/) and the Gene Expression Omnibus (GEO) database were used to obtain clinical data and gene expression profiles. Affymetrix Human Gene 2.0 ST Array was used to process the GSE17025 dataset. The level of TCGA gene expression was measured as Transcripts per million reads (TPM). The data of this study are from GEO and TCGA public databases.

### Identifications of differentially expressed genes (DEGs)

The GSE17025 expression profile was normalized and analyzed using R software and the limma package, while the TCGA EC dataset was normalized and analyzed using R software and the edge R package. Cut-off criteria were considered the |log_2_ Fold Change (log_2_FC)| >1 and adjusted *p*-value <0.05.

### Construction of WGCNA and module preservation

We applied the WGCNA to construct the gene co-expression network and identify the co-expression modules in R software [7]. In this study, we selected the minimum size (genome) 30 for the gene tree, the cutting line of 0.25 for the module tree, and combined some modules. We assessed how similar the module eigengenes (MEs) were, identified a cut line for the module dendrogram, and merged certain modules to better understand the module.

### Identification of clinically significant modules

Real hub genes were selected by drawing a Venn diagram which combining the module, DEGs in GEO database (*p* < 0.05, log_2_FC>1), and DEGs in TCGA database (*p* < 0.05, log_2_FC>1) together. The expression similarity of different samples is used to identify the WGCNA, while the relationship between the external clinicopathological information and the gene modules is used to identify clinically significant modules. Finally, gene modules that were highly correlated with certain clinical features were chosen as modules of interest for further analysis. Functional and pathway enrichment analysis of significant module was conducted according to the methods previously reported [13]. *P* < 0.05 was set as the cut off criterion.

### Construction and evaluation of the postmenopausal related prognostic model

LASSO regression analysis was performed to select essential prognostic genes by the “glmnet” R package. We listed the formula of the risk score for the predicting patients’ survival as follow:


$$\text{Risk score}=\sum {\text{n}}_{\text{i}}=\sum (\text{Coefi}\times {\text{x}}_{\text{i}})$$

Coefi represented the coefficient and x_i_ represented the expression level of hub genes. Subsequently, risk scores were calculated by the formula and gene expression for each patient. Then patients were divided into two groups (high- and low-risk) according to the median value of risk score. In these two subgroups, the clinicopathological features and gene expression profiles of each patient were displayed by "pheatmap" and "survival" R packages. The Kaplan-Meier survival analysis was conducted to compare the overall survival (OS) rate of the two subgroups by “survival” package of R (*P* < 0.05). The accuracy of the risk model was further evaluated by receiver operating characteristic (ROC) curve.

We validated possible uses of the predictive risk model and performed a survival analysis within our cohorts, and we obtained RNA-seq expression and clinical data from 30 patients that underwent surgery in the Department of Obstetrics and Gynecology, Nanjing First Hospital. Thirty samples without neoadjuvant therapy and who underwent surgical resection between January 2019 and December 2021 from patients in our center were selected. RNA isolation and reverse transcription-quantitative PCR were both conducted according to previously described methods [13]. The Institutional Ethics Committee (Human Research) of our hospital approved our research, and we obtained informed consent from all participants.

### *In vitro* validation of hub genes

Total protein of different types of tissues were extracted and used for western blot as described earlier [6]. Total RNA was extracted from tissues using TRIzol reagent (Tiangen, China). We used the PrimeScript RT-polymerase (Vazyme) to reverse-transcribe cDNAs to the mRNA of interest. Real-time quantitative RT-PCR (qRT-PCR) was conducted using SYBR-Green Premix (Vazyme) with specific PCR primers (Thermo Fisher Scientific, USA). GAPDH was taken as an internal control. The relative expression of the target gene was calculated using the 2^−ΔΔCt^ method. qRT-PCR was performed according to the manufacturer’s instructions.

### Statistical analysis

Statistical differences in the expression of hub genes in the normal and tumor samples were analyzed using a two-tailed student’s *t*-test in both the TCGA and GEO databases. All statistical tests and graphing were performed using R software and GraphPad prism 7.0. All analyses were conducted three times and represented data form three separate experiments. *P* value <0.05 was considered statistically significant.

## RESULTS

### Identification of DEGs in GEO and TCGA databases

**Figure 1:**
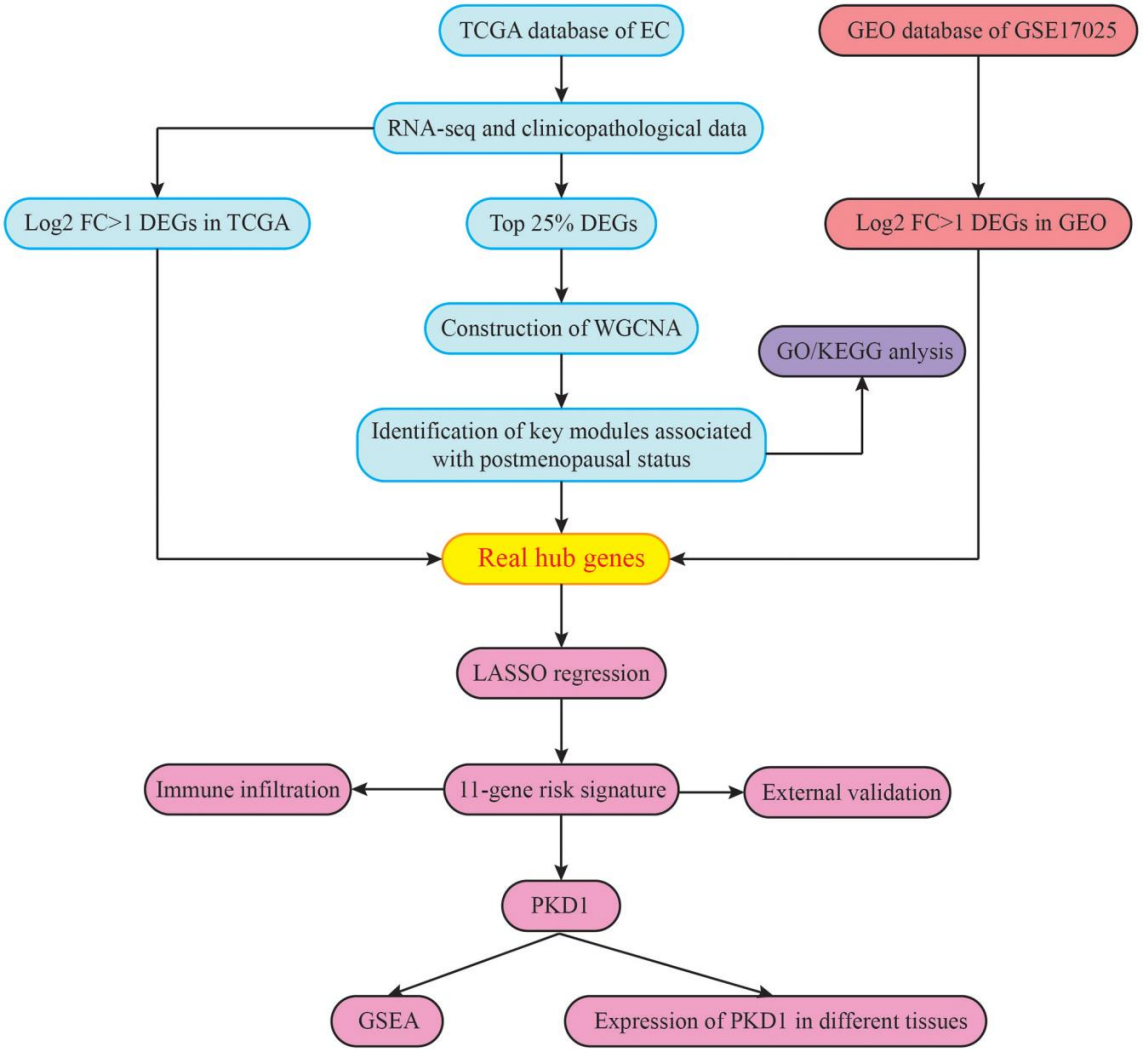
The flowchart of the study design.

**Figure 2:**
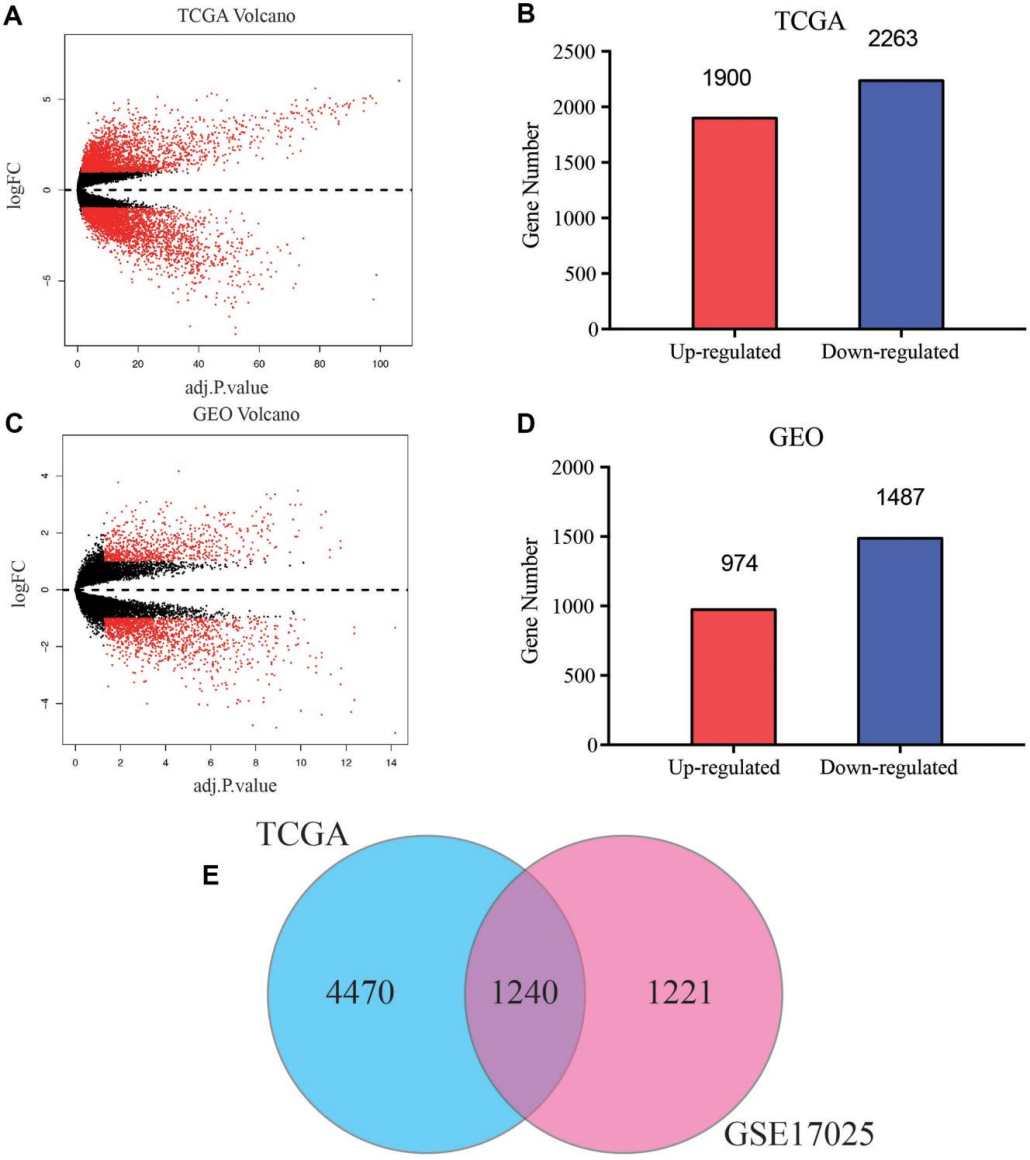
**Screening of common differentially expressed genes (DEGs) of TCGA and GEO databases.** (**A**) Volcano plot of DEGs between EC and normal endometrial samples in TCGA database. (**B**) Bar plot for DEGs of dysregulated genes in TCGA database. Red bar, up-regulated mRNA, blue bar, down-regulated mRNA. (**C**) Volcano plot of DEGs between EC and normal endometrial samples in GSE17025. (**D**) Bar plot for DEGs of dysregulated genes in GSE17025. (**E**) Venn diagram of DEGs between TCGA and GEO databases, the blue circle represents for total number of DEGs in TCGA, and the pink circle represents for total number of DEGs in GSE17025, the overlapped part is used for further analysis.

A brief study design was shown in Figure 1. The DEGs in both of the GEO and TCGA datasets were analyzed respectively using *p* < 0.05 and |log_2_FC| >1 as the cutoff criteria. The volcano maps of the DEGs in the two groups were constructed using the R package. Based on the screening guidelines, 4163 DEGs were acquired, consisting of 1900 up-regulated genes and 2263 down-regulated genes from EC tissues compared with normal endometrial tissues in TCGA dataset (Figure 2A, 2B). What’s more, we found a total of 2461 DEGs in the normal endometrial tissue in GSE17025, including 1487 downregulated and 974 upregulated genes (Figure 2C, 2D). In total, 1240 common genes are acquired by Venn diagram (Figure 2E).

### WGCNA and identification of key modules

**Figure 3:**
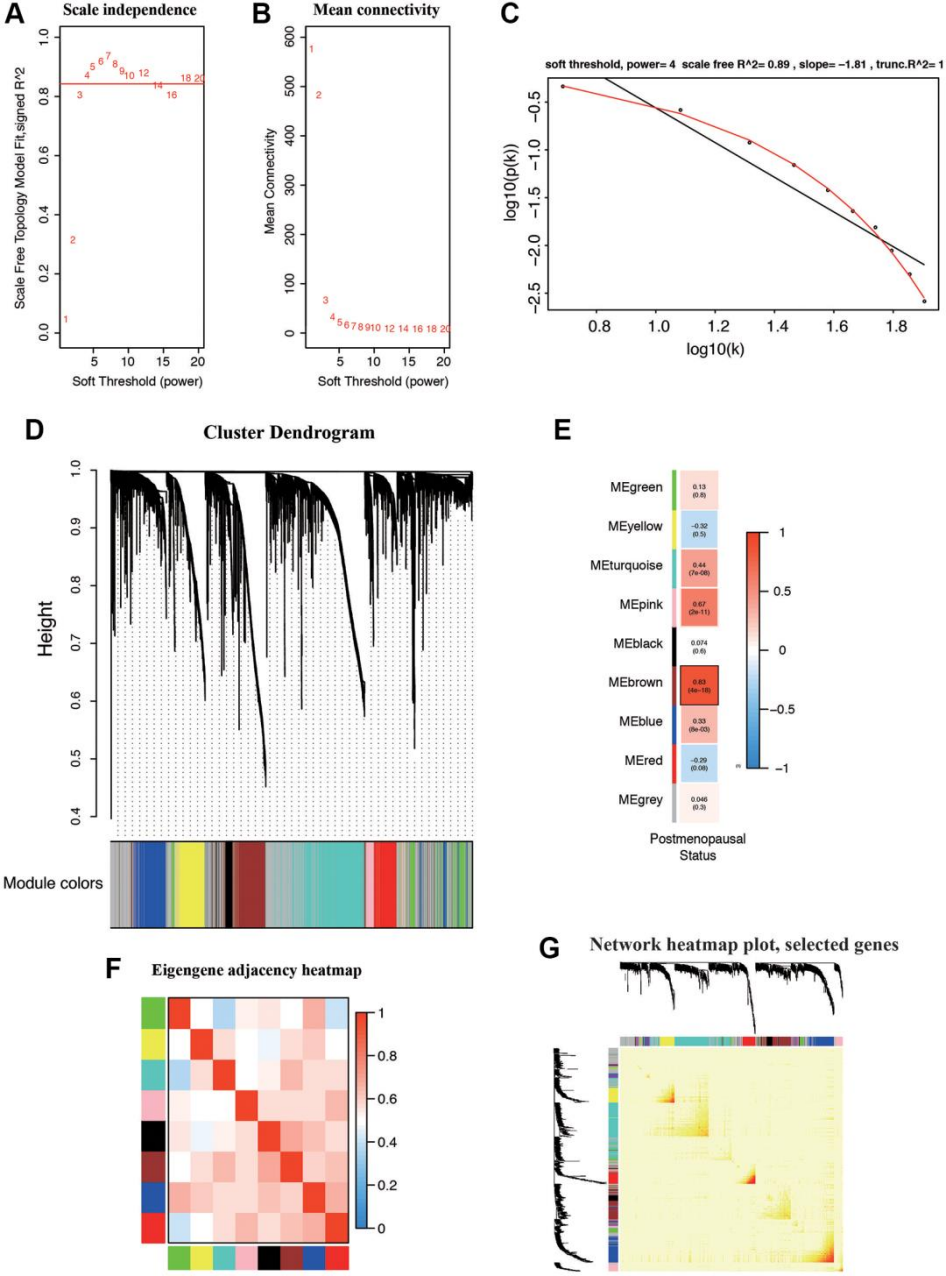
**Weighted gene correlation network (WGCNA) on the RNA-seq database and selection of hub genes.** (**A**, **B**) Screening and validation of the soft threshold. (**C**) Checking the scale free topology when β = 4. The x-axis demonstrates the logarithm of whole network connectivity, while the y-axis shows the logarithm of the corresponding frequency distribution. (**D**) Clustering dendrogram of common DEGs in EC tissues. (**E**) Correlation between modules and postmenopausal status. (**F**) Correlations between different modules. (**G**) Heatmap depicts the Topological Overlap Matrix (TOM) of genes selected for WGCNA. Light color represents lower overlap and deep color represents higher overlap.

We constructed co-expression network by TCGA dataset including 545 EC samples associated with whole clinical information. When constructing co-expression network with “WGCNA” software package, the expression value of the first 25% DEG is included. In our study, β = 4 (scale free R^2^ = 0.89) was selected as the soft-thresholding power to ensure a scale-free network (Figure 3A–3C). All selected genes were classified using the dissimilarity measure based on topological overlap matrix (TOM), which is based on the dynamic tree cutting algorithm and divides the tree into eight modules marked with different colors (Figure 3D). We identified the relationship between each module and the EC clinical information, with a particular emphasis on postmenopausal status (Figure 3E). Pearson’s correlation coefficient was used to assess the association between the co-expression modules in this study. Modules with various gene clusters were labeled in the topological overlap heatmap using different colors, with blue indicating a negative relationship and red indicating a positive relationship (Figure 3F). Then 1000 genes were randomly selected for the heatmap. Results of the clustering analysis indicated that the hierarchical clustering of the module eigengenes was representative of the modules. The dendrogram branches were clustered according to the eigengene relationships (Figure 3G).

First, modules with a greater MS were considered to have more connection with patients in postmenopausal status. However, most of the correlations were between low and moderate (R^2^ < 0.5), and the MS of the brown module (R^2^ = 0.83, *P* = 4e-18) was found to be higher than that of the rest of the modules. Therefore, the brown module with postmenopausal status was identified as the clinically significant module, which was extracted for further analysis.

### GO and pathway enrichment analysis of hub genes in brown module

**Figure 4:**
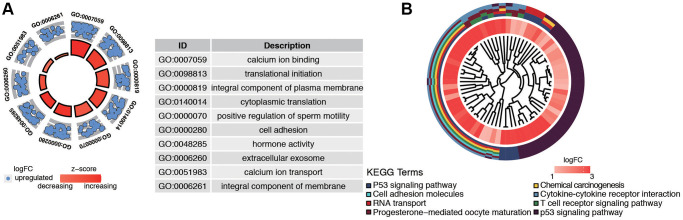
**Functional analysis of common DEGs from TCGA and GSE17025 datasets.** (**A**) GO analysis showing the differentially expressed postmenopausal related genes. (**B**) The significantly enriched pathways of the DEGs determined by KEGG analysis. Abbreviations: GO: gene ontology; KEGG: Kyoto Encyclopedia of Genes and Genomes.

GO and KEGG enrichment analysis were used to explore the function and pathways of the hub genes. The brown module, which consisted of 471 genes, was mainly associated with the following subclasses of GO classification (Figure 4A): calcium ion binding, translational initiation, integral component of plasma membrane, and cell adhesion. KEGG pathway analysis showed that top enriched terms were P53 signaling pathway, cell adhesion molecules, T cell receptor signaling pathway, and chemical carcinogenesis (Figure 4B). These suggested that brown module genes were closely related to the calcium channel and cell adhesion.

### Establishment and validation of a postmenopausal related prognostic signature

**Figure 5:**
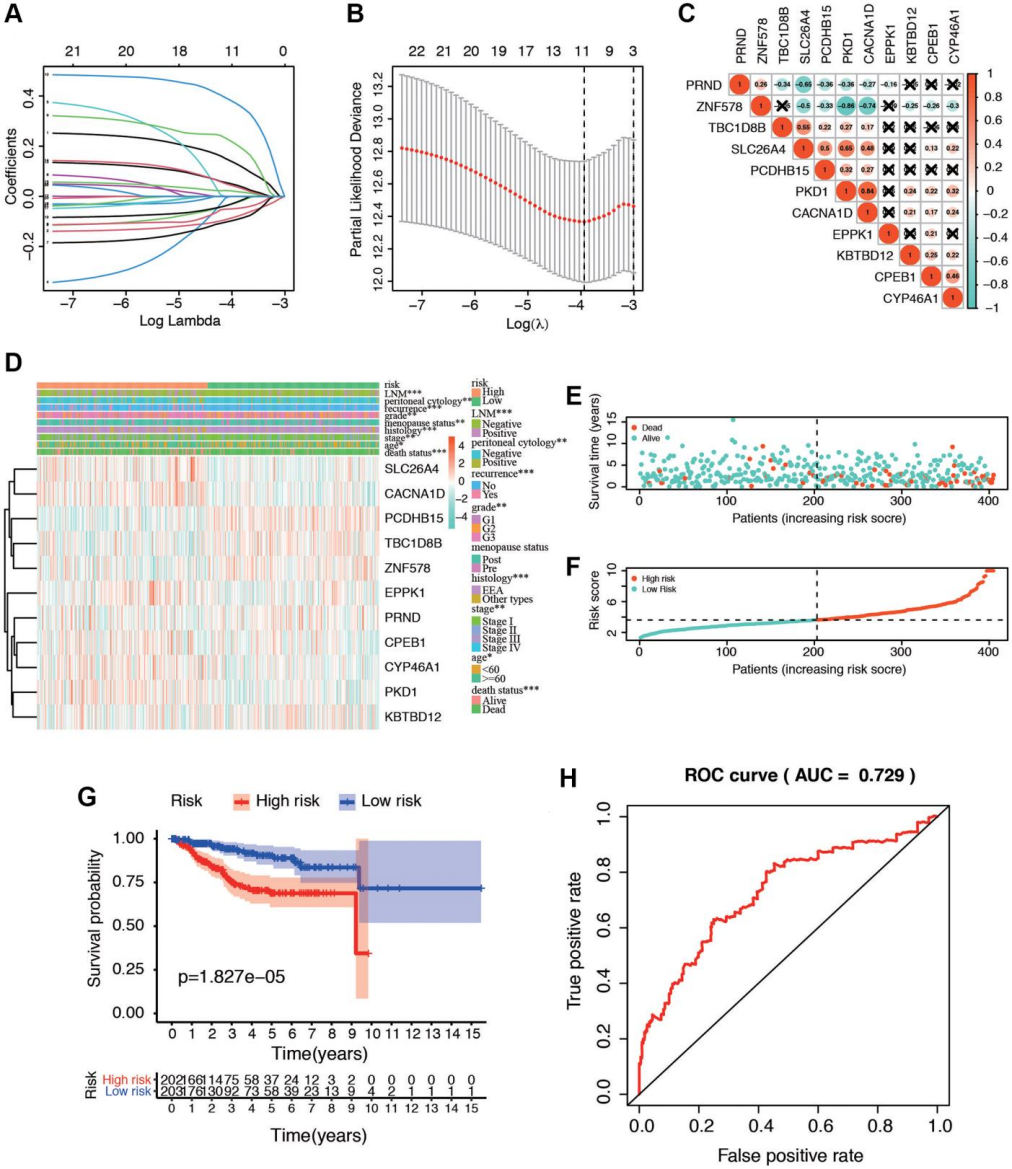
**Identification of postmenopausal related prognostic signature in EC patients.** (**A**) Plots of the ten-fold cross-validation error rates. (**B**) LASSO coefficient profiles of the eleven postmenopausal related genes. (**C**) Spearman correlation analysis with the selected 11 genes. (**D**) Heatmap and clinicopathological features of high- and low-risk groups. The samples are ordered by risk score, and the score decreases from left to right. (**E**, **F**) Risk score distribution in low- and high-risk groups. (**G**) Kaplan-Meier survival analysis of the low and high-risk group. (**H**) Time-dependent ROC curves for overall survival prediction for EC patients.

For screening out the potentially prognostic biomarkers for patients with postmenopausal status, LASSO regression was conducted. LASSO analyses identified 11 genes significantly associated with prognosis: PKD1, PCDHB15, PRND, TBC1D8B, ZNF578, SLC26A4, CACNA1D, CPEB1, CYP46A1, EPPK1, and KBTBD12 (Figure 5A, 5B). According to the results of LASSO regression analysis, we used the coefficients (Table 1) to construct the prognostic model as following: risk score = (PKD1 × 0.13192805) − (PCDHB15 × 0.082172079) − (PRND × 0.026889625) − (TBC1D8B × 0.093931462) − (ZNF578 × 0.048951829) + (SLC26A4 × 0.209421425) + (CACNA1D × 0.372518929) + (CPEB1 × 0.045936749) + (CYP46A1 × 0.039213661) + (EPPK1 × 0.019827739) − (KBTBD12 × 0.025230047). The patients were then ranked, in ascending order, according to the parameter, after which they were classified into low-risk and high-risk groups based on the median risk values. We further analyzed the relationship between the 11 genes (Figure 5C). We found that they were significantly relevant, especially between PKD1 and ZNF578, PKD1 and SLC26A4, CACNA1D and ZNF578, SLC26A4 and PRND. In addition, the expression of 11 genes in TCGA dataset in low-risk and high-risk patients was also confirmed in the heatmap. The expression profiles of the prognostic genes showed that PKD1, SLC26A4, CACNA1D, CPEB1, CYP46A1, and EPPK1 were highly expressed in the high-risk subgroup. We observed significant differences in the high- and low-risk groups related to peritoneal cytology, histology, menopause, tumor LNM, recurrence, grade, age, stage, and death status (Figure 5D). The dot plot displays the ranked risk score and survival status of each individual, with notable differences between the groups in terms of overall survival (OS) (Figure 5E, 5F). Kaplan-Meier curve analysis indicated that the OS of the high-risk group was significantly shorter than that of the low-risk group (*P* = 1.827e−05, Figure 5G). Analysis of the ROC curve indicated that the area under the ROC curve (AUC) of the prognostic HRGs model for OS was 0.729 (Figure 5H). These results demonstrated that the menopause-related risk signature had a high precision in the prediction of EC patients.

**Table 1:** Eleven postmenopause-associated genes and corresponding coefficient values.

**Metabolic associated gene**	**Coefficient**
PKD1	0.13192805
PCDHB15	−0.0821721
PRND	−0.0268896
TBC1D8B	−0.0939315
ZNF578	−0.0489518
SLC26A4	0.20942142
CACNA1D	0.37251893
CPEB1	0.04593675
CYP46A1	0.03921366
EPPK1	0.01982774
KBTBD12	−0.02523
Risk score	Low: <3.63
	High: ≥3.63

The risk signature mentioned above was further validated in the clinical cohort in our center (Supplementary Table 1). We conducted the RNA level sequencing of 30 EC patients and calculated the risk score of each patient with the formula mentioned above. EC patients in the cohort were divided into low- and high-risk groups based on the median risk score as before. Expression of PKD1 was compared between patients with pre- and postmenopausal status (Supplementary Figure 1A). Kaplan-Meier survival curves showed that patients with low risk scores had a longer OS, which was consistent with the predicted survival results of the risk model (*p* = 0.031) (Supplementary Figure 1B).

### Verification of expression of PKD1 in database and *in vitro*

**Figure 6:**
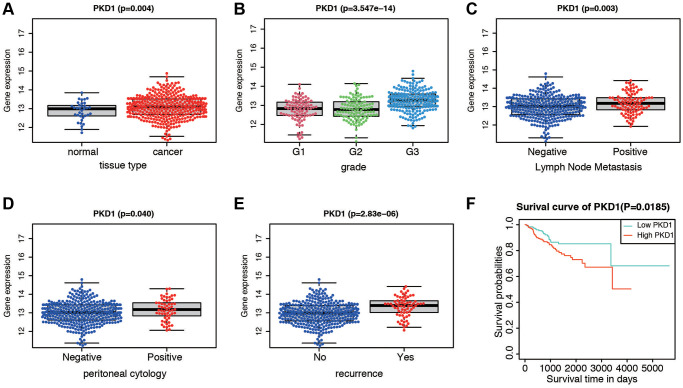
**Expression and survival validation of PKD1 in TCGA database.** Expression of PKD1 in different clinicopathological features. (**A**) Normal and cancer tissues. (**B**) Different grade. (**C**) Positive and negative lymph node metastasis. (**D**) Positive and negative peritoneal cytology. (**E**) Different recurrence status. (**F**) Kaplan-Meier survival plot of low and high expression of PKD1 according to the median value.

**Figure 7:**
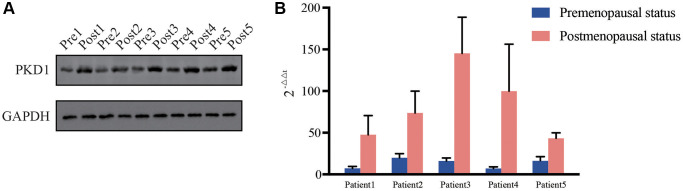
Relative expression of PKD1 in 5 pairs of pre- and post-menopausal patients in our center for (**A**) protein level and (**B**) RNA level. Pre, premenopausal status, Post, postmenopausal status.

We further validated the expression of PKD1 in different clinicopathological characteristics of EC patients in TCGA database. The results indicated that PKD1 was highly expressed in cancer tissue, grade 3, positive lymph node metastasis, positive peritoneal cytology, and recurrence groups (Figure 6A–6E). Patients were divided into two cohorts according to median value of PKD1. The results indicated that the expression of PKD1 was significantly associated with patient prognosis (Figure 6F). The analysis showed that PKD1 was statistically different in worse outcomes of EC patients. GSEA results showed that high expression of PKD1 was enriched in apoptosis, and low expression of PKD1 was enriched in B cell receptor signaling pathway, endometrial cancer, mTOR signaling pathway, and VEGF signaling pathway (Supplementary Figure 2). The immune infiltration was significant diverse in different expression of PKD1 (Supplementary Figure 3). To further explore the expression of PKD1 in different menopause status, we performed the qPCR and western blot (WB) validation in clinical specimens following the steps described above. As shown in Figure 7A, 7B, the expression levels of PKD1 in postmenopausal patients was more than that in the premenopausal patients in EC group. These results indicated that PKD1 may play a pivotal role in EC especially in postmenopausal women.

## DISCUSSION

The purpose of the present study was to screen out gene modules and hub genes that played a pivotal role in EC patients with postmenopausal status by WGCNA and LASSO analysis using the resources in TCGA and GEO databases. In this study, we identified a 471-gene module that had significant association with postmenopausal status by bioinformatics method and further revealed an 11-gene risk signature in this cluster. These risk model might act as an essential part in EC patients with postmenopausal status.

WGCNA had been used to distinguish related hub gene, biological pathway and tumor therapeutic target for complex diseases, such as familial genetic disease [14], Alzheimer’s disease [15], and many types of cancers [16, 17]. Co-expression network analysis as a powerful tool is also applied to study EC. Several modules and genes revealed by WGCNA analysis were reported to have an influence on pathological traits such as grade, stage, and prognosis [18, 19]. Other researches involved postmenopausal status belonged to clinical epidemiological investigation [20]. These studies either concentrated on pathological features associated genes or epidemiological characteristics of postmenopausal patients, but did not proclaim the mechanism that had an effect on postmenopausal EC patients.

According to the current literature, our findings identified the brown module, and further GO/KEGG analysis indicated that the biological mechanism of the brown module was closely associated with “calcium ion binding”, “P53 signaling pathway”, and “Cell adhesion molecules”. Calcium ion, especially calcium channel was correlated with risk and prognosis in EC [21]. Different cancers were linked to the expression of different calcium channels, such as TRPM7 in breast cancer [22] and TRPM4 in prostate cancer [23]. Previous study found that calcium ion and TRPV4 was required for calcium influx and contributes broadly to the development of endometrial cancer [24]. Calcium channel might also involve in tumorigenesis in postmenopausal EC patients. What’s more, p53 is thought to be an important tumor suppressor that influences multiple crucial biological processes, including apoptosis, cell-cycle arrest, and DNA repair [25]. More than 60% of patients with endometrial cancer develop TP53 mutations [12]; and mutant p53 proteins may not only abolish their tumor-suppressive functions, but also acquire oncogenic functions [26]. Cell adhesion could contribute to the invasive behavior of EC cells, and the underlying mechanism was related to TGFβ1-MEK-ERK1/2-integrin αvβ3 signaling pathway [27].

Protein kinase D1 (PKD1) is a serine threonine kinase and an important regulator of many kinase signal transduction pathways [28]. Previous studies have shown that PKD1 promoted breast cancer cell proliferation and estrogen independence [29, 30]. PKD1 is a serine/threonine kinase encoded by the PRKD1 gene [31], which can regulate various biological processes, including cell proliferation, survival, movement, Golgi tissue and membrane transport [32, 33]. In addition, endometrial cancer and breast cancer were both estrogen-mediated cancer. However, the underlying signaling mechanisms in EC are largely unknown. Some studies have shown that PKD1 stimulates NFκB, an important transcription factor involved in a variety of cellular mechanisms that plays a role in increasing the proliferation and growth rate of pancreatic cancer [34]. PKD1 had also been reported to be involved in the Notch signaling pathway [35]. During the transgenic model induced by KRAS12D, the expression of PKD1 contributes to the formation of precancerous lesions, indicating that PKD1 plays an essential role in the origination and progression of cancer cells [36]. Our study suggested that PKD1 was overexpressed in postmenopausal women compared with premenopausal women in protein and RNA levels. The mechanisms underlying PKD1 were associated with apoptosis, B cell receptor signaling pathway, endometrial cancer, mTOR signaling pathway, and VEGF signaling pathway. Together, these results suggest that PKD1 could be a potential therapeutic target in EC.

There were several risk signatures and biomarkers for predicting the prognosis of EC patients in different features aspects [37–39]. One study constructed a 5 cell cycle-related genes signature to evaluate prognosis of EC patients, and the AUC of 5-year survival reached to 0.733 [40]. A separate study found that gene signature models related to immunity in both EC and cervical cancer are effective at assessing patient prognosis and risk, which justifies future research into immunology [41]. However, as to the feature of postmenopausal status, there were no such risk models. Therefore, our signature was the first model associated with postmenopausal status and the validation proved to be highly precise in predicting OS in EC patients. Additionally, we used 30 patients from our center to validate the risk model. Our results demonstrate that this risk model is valuable for both clinical diagnosis and prognosis, while PKD1 could serve as a hub gene involved in EC progression.

To the best of our knowledge, there are no studies exploring the influence of menopausal status of EC, and this model helped us to recognize a new promising biomarker, PKD1 for EC from a clinical perspective. Additionally, this study reported a variety of bioinformatics methods that expose PKD1 as a novel therapeutic target for the treatment of EC patients, and validated with our own samples in RNA and protein levels, highlighting the potential of this molecular for therapeutic candidate discovery.

However, there are several limitations in our study. First of all, this is a retrospective study, and this risk signature is developed from two online databases, but not our own samples, and this risk model should be further validated by large-group sequencing. Secondly, our study assesses menopause in EC patients, though other clinicopathological characteristics are important during oncological pathologies. These must be integrated and studied to better understand tumorigenesis and its progression in EC patients. Lastly, the specific mechanism regulating PKD1 in EC cells must be further studied both *in vitro* and *in vivo*, and how it affects metastasis in EC patients requires further attention.

## CONCLUSION

In conclusion, we performed a WGCNA approach integrating TCGA and GEO databases, and constructed a gene co-expression network to reveal a postmenopausal specific module and potential molecular mechanism underlying the tumorigenesis for EC. Furthermore, an 11-gene signature is constructed and validated. Finally, PKD1 was identified as potential biomarker that played a key role in the progression of EC with postmenopausal status.

## Supplementary Materials

Supplementary Figures

Supplementary Table 1
