# Supplementary material for: Integrated bioinformatics data analysis reveals a risk signature and PKD1 induced progression in endometrial cancer patients with postmenopausal status
Source: Aging (Albany NY). 2022 Jul 9;14(13):5554–70. doi: 10.18632/aging.204168 (PMC9320543; doi:10.18632/aging.204168)
Supplement: Supplementary Figures [file aging-14-204168-s001.pdf]

SUPPLEMENTARY FIGURES

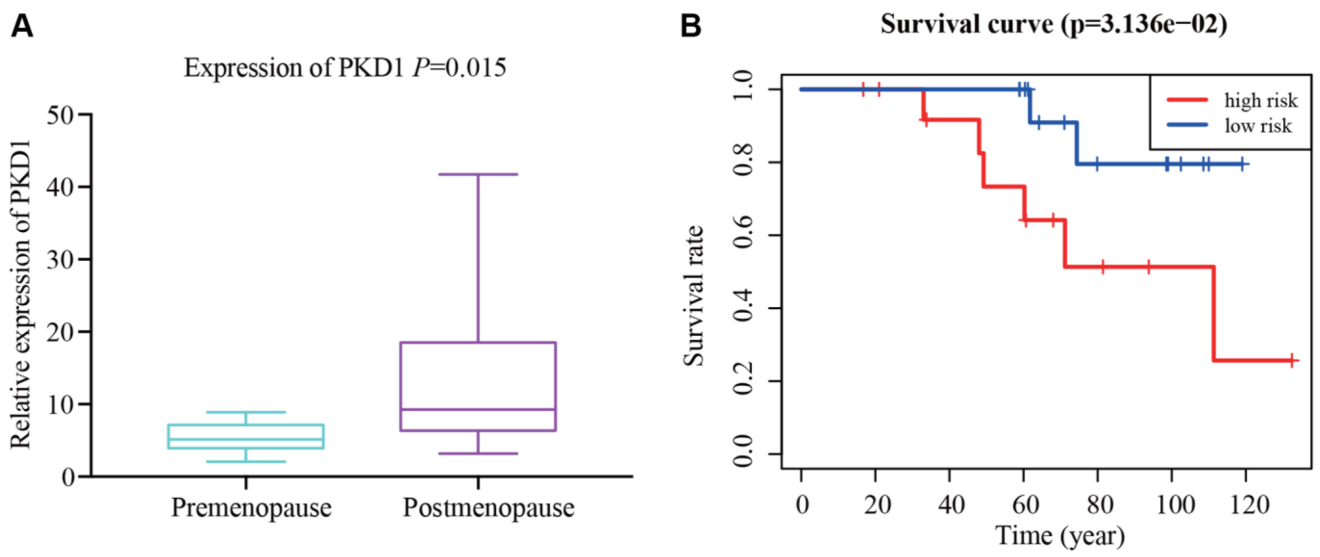

Supplementary Figure 1. Validation of the risk model. (**A**) Expression of PKD1 (**B**) Survival curve of patients in our center.

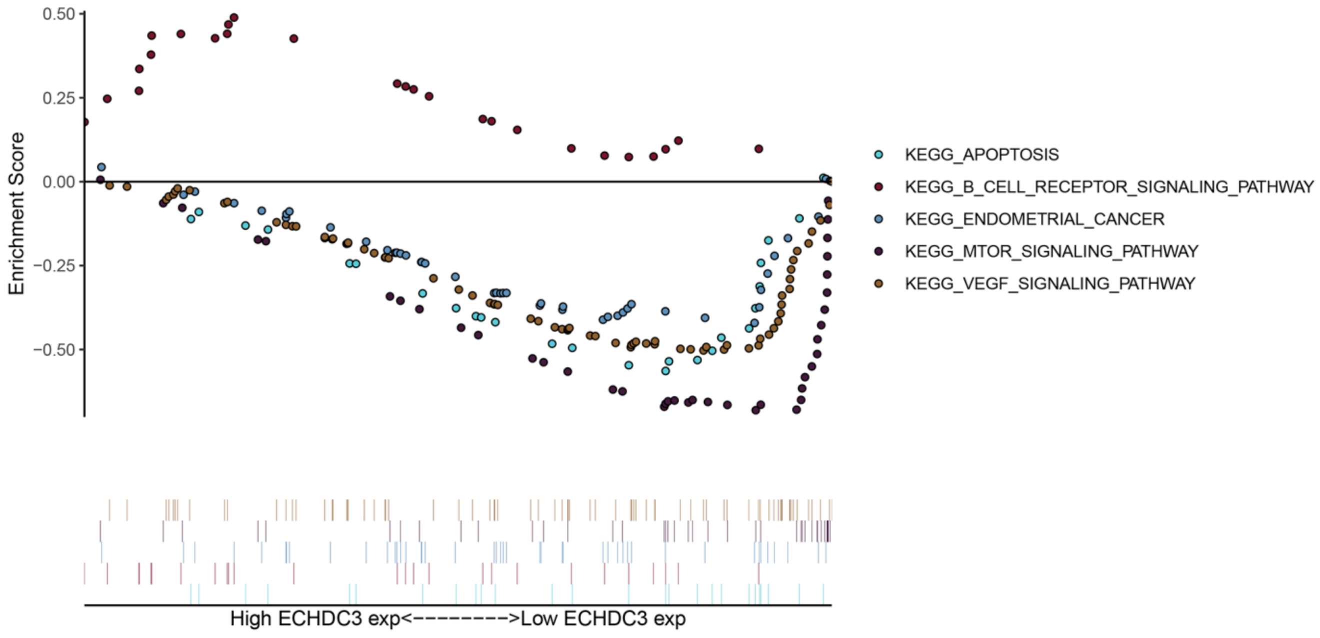

Supplementary Figure 2. KEGG analysis of gene set enrichment analysis (GSEA) in high- and low- expression of PKD1 groups.

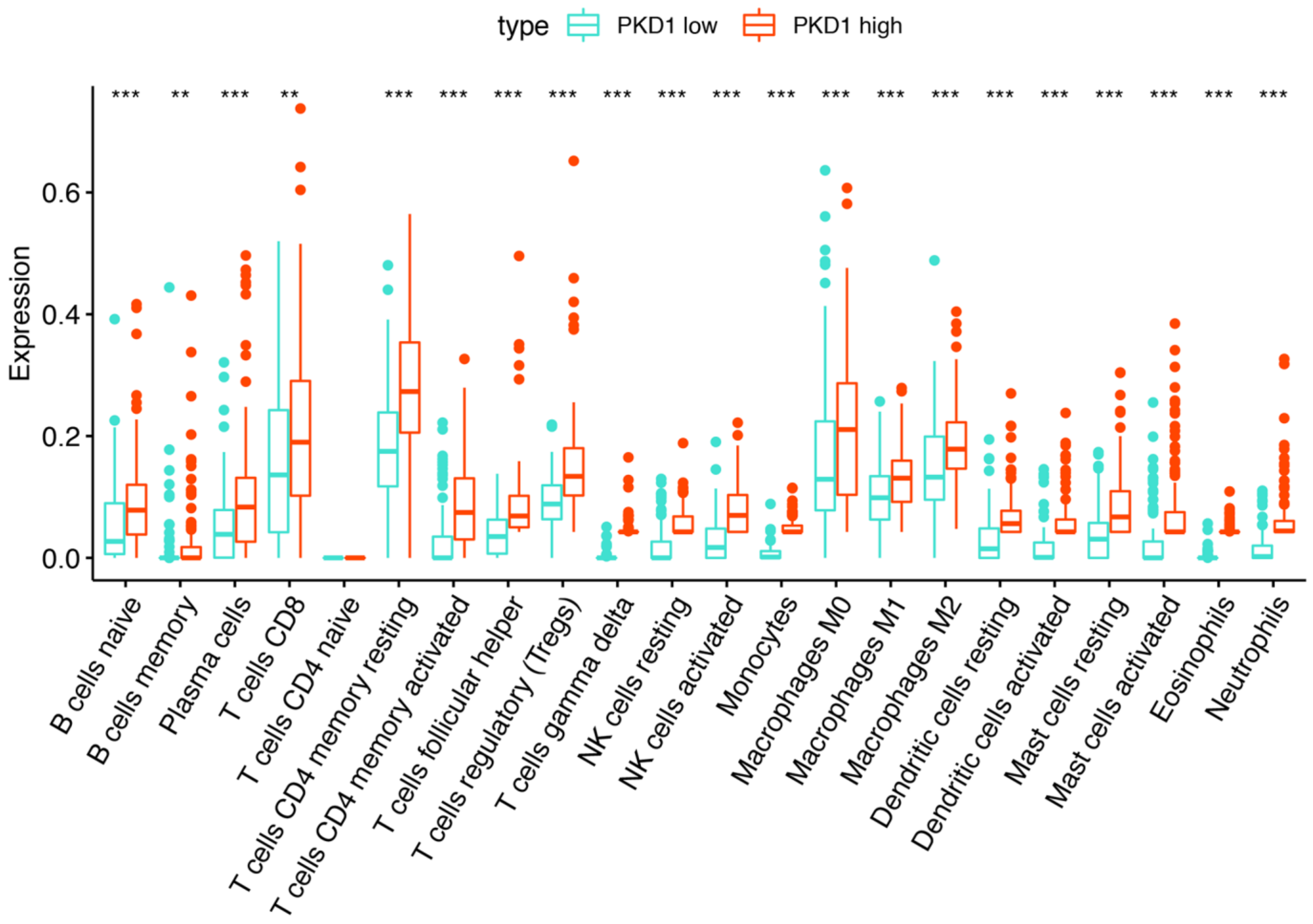

Supplementary Figure 3. Analysis of 22 immune cell in low- and high-expression of PKD1 groups.
