# Supplementary material for: Integrated bioinformatics data analysis reveals a risk signature and PKD1 induced progression in endometrial cancer patients with postmenopausal status
Source: Aging (Albany NY). 2022 Jul 9;14(13):5554–70. doi: 10.18632/aging.204168 (PMC9320543; doi:10.18632/aging.204168)
Supplement: Supplementary Table 1 [file aging-14-204168-s002.pdf]

SUPPLEMENTARY TABLE

Supplementary Table 1. Clinicopathological baseline of 30 patients in our center.

**Variables**	***N* (%)**
Age	
<60	18 (66.7)
>60	12 (33.3)
Grade	
1–2	19 (63.3)
3	11 (36.7)
FIGO stage	
I	17 (56.7)
II–IV	13 (43.3)
Menopausal status	
Pre-menopause	13 (43.3)
Post-menopause	17 (56.7)
Recurrence	
Recurrence-free	15 (50.0)
Recurrence	15 (50.0)
Myometrial invasion	
Negative	18 (66.7)
Positive	12 (33.3)
Living status	
Alive	22 (73.3)
Dead	8 (26.7)
